# Editorial: Brain injury and repair following cerebrovascular diseases: From bench to bedside

**DOI:** 10.3389/fncel.2022.986969

**Published:** 2022-08-12

**Authors:** Gaiqing Wang, Qifu Li, Anwen Shao, Yue He, Lei Huang, Dirk M. Hermann

**Affiliations:** ^1^Department of Neurology, Sanya Central Hospital (Hainan Third People's Hospital), Hainan Medical University, Sanya, China; ^2^Department of Neurology, First Affiliated Hospital of Hainan Medical University, Haikou, China; ^3^Department of Neurosurgery, The Second Affiliated Hospital of Zhejiang University School of Medicine, Hangzhou, China; ^4^Department of Neurosurgery, Tongji Hospital Tongji Medical College, Huazhong University of Science and Technology, Wuhan, China; ^5^Department of Neurosurgery, Loma Linda University School of Medicine, Loma Linda, CA, United States; ^6^Department of Physiology and Pharmacology, Loma Linda University School of Medicine, Loma Linda, CA, United States; ^7^Department of Neurology, University Hospital Essen, University of Duisburg-Essen, Essen, Germany

**Keywords:** self-repair system, cerebrovascular disease, brain injury, clearance system, phagocytosis, inflammation

Endogenous attempts of self-repair after cerebrovascular disorders are complex. Brain remodeling processes can therapeutically be stimulated. Underlying cellular and molecular mechanisms remain insufficiently characterized. This issue aims to clarify mechanisms of brain injury and endogenous repair in cerebrovascular disorders.

Clearance of waste products from the brain is important for self-repair under conditions of cerebrovascular injury. In their review, Jiachen et al. addressed the structural composition and main components of clearance systems in the brain, which include the glymphatic system and the meningeal lymphatic system that closely interact with neural cells, such as astrocytes and microglia, to carry out vital clearance functions. In another review on drainage systems, Sichao et al. concluded the glymphatic system plays a dual role in the process of cerebral edema after stroke with a harmful role in the early stage of edema formation and a beneficial role during edema resolution. The function of glymphatic system is supported by astrocytic AQP4. In another review, Rentang et al. discussed the dual roles of microglia in the brain after intracerebral hemorrhage (ICH). M1 microglia are widely considered as the deleterious phenotype which arouse acute inflammatory responses, oxidative stress, excitotoxicity, and cytotoxicity to cause neuron death, while M2 microglia is regarded as the beneficial phenotype which inhibits inflammation, clears hematoma and promotes tissue regeneration. Jiaxin et al. reviewed the mechanistic signaling pathways of erythrophagocytosis and highlights the potential of harnessing macrophage (Mϕ) mediated phagocytosis for hematoma removal. This process is mediated by scavenger receptors and the upstream regulators for erythrophagocytosis such as PPARγ, Nrf-2, AMPK and others. Linqian et al. discussed the effects and mechanisms of oxidative stress after ICH and its relationship with inflammation and autophagy, as well as the current state of antioxidant therapies in ICH.

In an original article, Bing et al. investigated the role of mast cells (MCs) in subarachnoid hemorrhage (SAH) and showed that MC activation contributed to brain edema and neurological impairment after SAH. Furthermore, MC-derived tryptase exacerbates microglia-related neuroinflammation by interacting with microglial PAR-2. In another original article, Weijia et al. showed that L-F001 was an efficient antioxidant and ferroptosis inhibitor, which significantly restored RSL3-induced broken iron homeostasis, reduced lipid peroxidation, and JNK overactivation in HT22 cells.

Jie et al. reviewed the relationship between ICH and sepsis, which were mutually exacerbated via similar pathophysiological mechanisms, which involved systemic inflammation and vascular dysfunction. Zeyu et al. discussed the role of gut microbiota post stroke. They highlighted the possible utility of gut microbiota as a therapeutic target in ischemic stroke.

Shuo-Qi et al. summarized the role of sphingosine-1-phosphate (S1P) signaling in brain ischemia with specific focus on inflammation and immune responses, and discussed the current and future perspectives of targeting S1P for ischemic stroke treatment. Shuiping et al. reviewed the effects of low-frequency transcranial ultrasound stimulation (TUS) on ischemic brain injury, with specific focus on mechanical actions, microvascular flow, thrombus resolution, as well as infarct volume reduction post stroke. Hui et al. summarized the effects of physical exercise on ischemic injury with specific focus on blood-brain barrier integrity, angiogenesis, neuroprotection, and functional neurological recovery. Rui et al. evaluated evidence on the therapeutic potential of remote ischemic conditioning (RIC) under conditions of vascular cognitive impairment (VCI), with specific focus on blood pressure control, secondary stroke prevention, cerebral blood flow, microvascular integrity, white matter remodeling, oxidative stress, and brain inflammatory responses. RIC was judged to be a potential promising treatment of VCI.

In summary, the contributions of this Research Topic provide a timely and comprehensive overview on current research activities regarding mechanisms of brain injury and self-repair in the cerebrovascular field. Novel mechanisms and disease targets were identified. Preclinical studies pointed out the possible clinical potential of therapeutic interventions. Yet, the clinical utility of these strategies requires further scrutiny, before studies in human patients can be considered. As editors, we would like to thank the authors for their contributions. We hope that this Research Topic provides a useful stimulus to the readers for subsequent studies, which might pave the way for clinical studies in humans.

## Author contributions

All authors listed have made a substantial, direct, and intellectual contribution to the work and approved it for publication.

## Funding

This work was supported by Hainan Province Clinical Medical Center, National Natural Science Foundation of China (Nos. 81771294; 82160237), Natural Science Foundation of Hainan Province (No. 822MS210) and German Research Foundation (DFG; Nos. 389030878, 405358801, to DMH).

## Conflict of interest

The authors declare that the research was conducted in the absence of any commercial or financial relationships that could be construed as a potential conflict of interest.

## Publisher's note

All claims expressed in this article are solely those of the authors and do not necessarily represent those of their affiliated organizations, or those of the publisher, the editors and the reviewers. Any product that may be evaluated in this article, or claim that may be made by its manufacturer, is not guaranteed or endorsed by the publisher.

